# Development of a new Sonovue™ contrast-enhanced ultrasound approach reveals temporal and age-related features of muscle microvascular responses to feeding

**DOI:** 10.1002/phy2.119

**Published:** 2013-10-27

**Authors:** William Kyle Mitchell, Bethan E Phillips, John P Williams, Debbie Rankin, Kenneth Smith, Jonathan N Lund, Philip J Atherton

**Affiliations:** 1 Division of Clinical Metabolic and Molecular Physiology, School of Graduate Entry Medicine and Health, University of NottinghamDerby, U.K.; 2 Department of Anaesthesia, Royal Derby HospitalDerby, U.K.; 3 Department of Surgery, Royal Derby HospitalDerby, U.K.

**Keywords:** Aging, CEUS, microcirculation, muscle, nutrition

## Abstract

Compromised limb blood flow in aging may contribute to the development of sarcopenia, frailty, and the metabolic syndrome. We developed a novel contrast-enhanced ultrasound technique using Sonovue™ to characterize muscle microvasculature responses to an oral feeding stimulus (15 g essential amino acids) in young (∼20 years) and older (∼70 years) men. Intensity-time replenishment curves were made via an ultrasound probe “fixed” over the quadriceps, with intermittent high mechanical index destruction of microbubbles within muscle vasculature. This permitted real-time measures of microvascular blood volume (MBV), microvascular flow velocity (MFV) and their product, microvascular blood flow (MBF). Leg blood flow (LBF) was measured by Doppler and insulin by enzyme-linked immunosorbent assay. Steady-state contrast concentrations needed for comparison between different physiological states were achieved <150 sec from commencing Sonovue™ infusion, and MFV and MBV measurements were completed <120 sec thereafter. Interindividual coefficients of variation in MBV and MFV were 35–40%, (*N* = 36). Younger men (*N* = 6) exhibited biphasic vascular responses to feeding with early increases in MBV (+36%, *P* < 0.008 45 min post feed) reflecting capillary recruitment, and late increases in MFV (+77%, *P* < 0.008) and MBF (+130%, *P* < 0.007 195 min post feed) reflecting more proximal vessel dilatation. Early MBV responses were synchronized with peak insulin but not increased LBF, while later changes in MFV and MBF occurred with insulin at post absorptive values but alongside increased LBF. All circulatory responses were absent in old men (*N* = 7). Thus, impaired postprandial circulation could impact age-related declines in muscle glucose disposal, protein anabolism, and muscle mass.

## Introduction

Aging is the main risk factor for cardiovascular disease (Hayflick 2007) and age-related declines in blood flow to limbs could play a substantial role in a number of non-communicable diseases, for example, the metabolic syndrome (Lind and Lithell 1993; Goodwill and Frisbee 2012) and sarcopenia/frailty (Mitchell et al. 2012). In older age, whole-limb blood flow, adjusted for lean mass, is reduced (Dinenno et al. 1999), due to both a decrease in capillary numbers (Coggan et al. 1992) and increased sensitivity to vasoconstrictors, that is, α-adrenergic innervation and the renin-angiotensin aldosterone systems (Barrett-O'Keefe et al., 2013b). Moreover, aging causes endothelial dysfunction which compromises responsiveness to vasodilatory stimuli (Celermajer et al. 1994; Seals et al. 2006). For example, exercise-associated increases in limb blood flow are diminished in older age (Donato et al. 2006), potentially compromising O_2_ delivery, metabolite clearance and performance. Nutrient intake also increases whole-limb perfusion (Raitakari et al. 2000), another vasodilatory effect attenuated with age (Skilton et al. 2005). This could impact glucose, insulin, and amino acid delivery and thus impair aspects of postprandial homeostasis for example, muscle glucose disposal and protein anabolism (Phillips et al. 2012). Further interest in the physiological significance of age-related vascular dysfunction has been generated by recent data suggesting that these are not inevitable aspects of the aging process but modifiable factors (Najjar et al. 2005; Seals et al. 2006) with potential interventions including resistance exercise training, which rejuvenates acute leg-flow responses to feeding and exercise (Phillips et al. 2012). Although being important, these observations, in keeping with most studies that describe age-related changes in hemodynamic responses, have mainly relied upon methods that do not differentiate between vessels types nor address the separate elements of blood flow, that is, *blood volume* and *velocity*, which together comprise perfusion.

The functional organization of muscle microvasculature has come to be understood in terms of a “microvascular unit” (MVU), with a single terminal arteriole giving rise to ∼20 capillaries, each ∼1 mm long, which run along fibers in both directions toward collecting vessels. Thus, microvascular flow is axial with capillaries parallel to muscle fibers (Bloch and Iberall 1982; Delashaw and Duling 1988). Typically, two thirds of capillaries are empty at a given time in resting skeletal muscle (Honig et al. 1982) and muscle blood flow is ∼3 mL min^−1^ 100 g^−1^; only 3–5% of that seen in visceral organs (Sweeney and Sarelius 1989; Frame and Sarelius 1993; Sarelius 1993). In response to hyperemic stimuli, vasomotion ceases as terminal arterioles dilate (Segal 2005). The dilatation of these arterioles causes a rapid increase in perfused capillary density and thus the fraction of tissue volume made up of blood within the microvasculature; this is known as the microvascular blood volume (MBV). Postprandial insulinemia mediates muscle capillary recruitment, doubling MBV, at least in the young, (Rattigan et al. 1997; Vincent et al. 2004; Sjøberg et al. 2011) and increasing surface area for insulin, glucose, and amino acids (AA) to leave the circulation and contact myocytes, thus aiding glucose disposal and insulin/AA-mediated protein anabolism (Wilkes et al. 2009). Remarkably, changes in MBV can occur with little change in total limb blood flow (Krogh 1919; Honig et al. 1980; Sweeney and Sarelius 1989). With increasing intensity of hyperemic stimulus, blood velocity and limb flow increase with little further change in perfused capillary density; such changes are attributed to an upstream shift in the regulation of flow, with vasodilatation encompassing intermediate and proximal arterioles and feed vessels to increase bulk flow (Segal 2005).

It has recently been recognized that contrast-enhanced ultrasound (CEUS) has the ability to assess MBV in real-time, as well as the velocity of blood flow within the microvasculature, (the microvascular flow velocity, MFV) and thus their product (the microvascular blood flow (MBF), with units of volume of blood per volume of tissue per unit time) (Wei et al. 1998). CEUS depends upon measurement of the acoustic backscatter of intravascular capsule-stabilized gas-filled microbubbles. While CEUS is widely used in diagnostic imaging, its use in circulatory research remains relatively limited (Quaia 2005). However, CEUS has been used to address aspects of muscle microvascular function in response to exercise (Dawson et al. 2002; Krix et al. 2010), insulin (Vincent et al. 2002; Clerk et al. 2004) and disease (Womack et al. 2009; Amarteifio et al. 2011). A range of microbubble preparations and detection methods have been used with most reporting on measurement of MBV or ΔMBV/Δt, without assessing MFV or MBF. In a 13-min protocol, Sjøberg (et al. 2011) recently described a method combining intermittent high-mechanical index (MI) pulses which achieved microbubble disruption and thus measurement of MFV, with real-time, continuous low MI recording that allowed precise assessment of MBV. However, due to restricted international availability of the contrast agent used for these studies, there was a need to develop and validate application of a new contrast agent for these purposes. Thus, our objectives were: (1) to validate a new rapid and clinically acceptable, noninvasive high-resolution technique to quantify separate elements of blood flow within muscle microvasculature and, (2) to apply this method to interrogate the effects of aging on muscle microvascular responses to feeding.

## Methods

**Figure 1:**
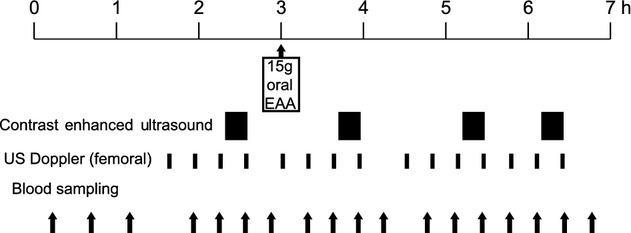
Protocol for the measurement of microvascular blood volume (MBV) and microvascular flow velocity (MFV) by contrast-enhanced ultrasound (CEUS) and leg blood flow (LBF) by femoral arterial US Doppler before and after a 15 g EAA feed.

All human volunteers gave written informed consent. The study was approved by the University of Nottingham Medical School Ethics Committee (F/3/2009) and conformed to the Declaration of Helsinki. Thirty-six healthy male volunteers aged (18–28: mean 20 years; or 65–75 years: mean 70 years) underwent CEUS assessment of quadriceps muscle blood flow using Sonovue™ (Bracco, Milan, Italy) SF_6_ microbubbles delivered by continuous infusion following an intermittent brief high MI/continuous low MI protocol. Recruitment was via standard letter to addresses proximate to the research center. Exclusion criteria included diabetes mellitus, cardiovascular disease, and mass index index (BMI) <18 or >28. Volunteers fasted 12 h preceding studies. A subgroup of six young and seven older men went on to receive a 15 g oral mixed essential amino acid (EAA) feed (Histidine 1.21 g, Isoleucine 1.73 g, Leucine 3.59 g, Lysine 3.07 g, Methionine 0.95 g, Phenylalanine 0.91, Tryptophan 1.13 g, Threonine 0.48 g and Valine 1.86 g).This dose provides a modest, reliable insulin secretion response. CEUS recordings were made 45, 135, and 195 min after feeding to determine temporal responses to feeding and effects of age. Timepoints were chosen to resolve microvascular flow before (0–90 min), during (90–180 min), and after (180+ min) the expected period of stimulated muscle protein synthesis that follows EAA ingestion. Recordings were made in a temperature-controlled clinical room with minimal variation in sensory stimulation. Volunteers remained supine for 90 min prior to commencement of the protocol and throughout. Plasma insulin concentrations were measured throughout via enzyme-linked immunosorbent assay (DRG Instruments GmbH, Marburg, Germany). Dual-energy X-Ray absorptiometry (DXA; Lunar Prodigy II, GE Medical Systems, Little Chalfont, Buckinghamshire, U.K.) was used to determine body composition. Common femoral arterial blood flow was measured by ultrasound (US) Doppler using a Phillips iU22 scanner (Santa Ana, CA) and a 9-3 MHz linear-array transducer (Phillips L9-3) in “Vascular Arterial” mode with averaging over 12 repeats, each including an number of complete cardiac cycles measured over ∼6 sec, distributed across a period of 45 min before and 15 min after CEUS recordings, Figure 1.

### Features of Sonovue™ microbubbles

Sonovue™ is provided as 25 mg of lyophilized powder in an SF_6_ atmosphere, which upon reconstitution with 5 mL 0.9% saline, and 20 sec of vigorous shaking, forms a dispersion with ∼2 × 10^8^, or 8 μL, of SF_6_ microbubbles mL^−1^. The mean microbubble diameter is 2.5 μm; 90% are <6 μm and 99% <11 μm. The median effective half-life at relevant doses is 0.97 min in men and 1.23 min in women (Morel et al. 2000). Using the equation C = Q/KV, where C is the steady-state concentration of microbubbles, Q the infusion rate, K the rate constant, and V the volume of distribution (plasma volume), assuming Q = 2 × 10^8^ bubbles/min, K = 0.71, V = 3 L, C = 93,000 bubbles/mL of plasma. Perflutren, for comparison, achieves a microbubble concentration more than eightfold higher (Sjøberg et al. 2011). There is a linear relationship between the acoustic intensity (AI) generated by resonating microbubble and their concentration (Wei et al. 1998). Sonovue™ has been shown to have minimal impact on rheology even in capillaries, without a tendency to clump or block exchange vessels (Schneider et al. 2012). Microbubbles resonate at harmonics of the insonated, or *fundamental* frequency, allowing scanners to detect signal from microbubbles, while suppressing signal reflected by tissue. Most of the signal reflected off tissue is close in frequency to the fundamental. By high-pass filtering, the reflected wave of this signal is suppressed making tissue appear dark while sensitive registration of harmonics, predominantly reflected by intravascular microbubbles, makes even small vessels appear white (Quaia 2005).

### Experimental protocol

The ultrasound transducer was secured onto the thigh using a polyethylene-housing unit (manufactured by University of Nottingham's Medical Engineering) with circumferential Velcro™ straps (Amsterdam, NL), which maintained constancy of position throughout the experiment. CEUS imaging of the quadriceps (predominantly m. vastus lateralis [VL] and intermedius) was performed using a Phillips iU22 scanner and L9-3 probe. All recordings were made in Contrast Mode, which acts to minimize tissue-dependent signal while maximizing the proportion of recorded signal that is reflected off insonated microbubbles by selective register of harmonics (Quaia 2005). Cine recordings were made at 21 Hz with low persistence (to maximize temporal resolution), contrast resolution C30 (simple harmonic registration), gain 95%, cursor gain constant throughout, depth 4 cm, focused 2–4 cm, and a working MI of 0.08 (MI is a unitless index proportional to peak negative pressure **×** transmit frequency^−0.5^), causing microbubbles to resonate without destruction. Sonovue™ microbubbles were reconstituted using normal saline. Typical recovery from the transfer system (mini-spike plus; Bracco) was 4.8 mL, which was increased to 5 mL using normal saline, again washed via the transfer system to increase yield. Microbubbles were delivered via a Vueject oscillating infusion pump (Bracco) and a low volume PVC connection line (Acist, Eden Prarie, MN) to an 18GA (1.3 mmØ) cannula sited in the left median cubital vein and infused at a priming rate of 2 mL min^−1^ for 1 min followed by 1 mL min^−1^ for 3 min, thus one vial of Sonovue™ provided adequate volume for a 4-min recording period. Steady concentration of microbubbles in muscle vasculature was achieved within ∼2 min. Measurements of MBF were made between 2.5 and 4 min after commencement of infusion. Based on the technique described by Sjøberg et al., a continuous, real-time, low MI recording was made of microbubble replenishment following brief, intermittent high MI “flashes” (MI 1.12, duration 0.57 sec). Three successive, back-to-back, 30-sec recordings were made of flash/replenishment cycles. The recording protocol was thus completed in 90 sec. See Video S1 included in supplementary on-line content. Cine recordings were exported to quantification software (Q-Lab, Philips), which permits the selection of regions of interest (ROI) that can be constructed to minimize signal contribution from connective tissue and large vessels and maximize included muscle. The echogenicity of the ROI is calculated as an AI, usually expressed as its acoustic index, a logarithmic transform with units dB. However, as this precludes subsequent arithmetic manipulations including subtraction of a background, nonmicrobubble echogenicity, AI was maintained as a linear quantity throughout.

### Achieving a steady-state concentration of microbubbles in skeletal muscle vasculature and demonstration of total microbubble destruction

In order to derive parameters of flow using this technique it is necessary to demonstrate that a steady state exists in the circulation whereby muscle microvascular concentration of microbubbles is constant. Continuous recording of muscle AI was made from the commencement of microbubble infusion. AI was averaged across individuals within age groups. AI recorded during the last 20 sec before initiation of the high MI flash-replenishment recordings was subjected to linear regression analysis to establish mean dAI/d*t* and its 95% confidence interval (CI). Furthermore, to demonstrate that constancy of microbubble concentration continued throughout the recording period, the AI recorded in each individual during the 1 sec prior to the second and third high MI flashes were compared. In order to demonstrate effective and consistent microbubble destruction, the size of the decrement in AI upon the first high MI flash was compared to the size of the increment that occurred upon infusion.

### Calculation of microvascular flow parameters

A period immediately post flash was used to calculate the background AI, attributable to tissue echogenicity and rapidly filling larger, nonexchange vessels. Following the work of Sjøberg et al., the period chosen was c. 0.5-sec long (from 0.048 to 0.52 sec post flash, using 10 measurements, made at 21 Hz). The mean AI during this period was calculated and subtracted from all subsequent values during that replenishment curve. The mean AI across three repeated flash-replenishment recordings was calculated after background correction and was truncated at 28 sec. Mean AI was expressed against time and, according to the method of Wei et al. (1998) was fitted to the exponential function of one phase association, *y* = *y*_0_ + A(1−e^−β*t*^). Due to background correction, *y* = A(1−e^−β(*t*−B*t*)^) where B*t* ≈ 0.28 sec, the midpoint of the time period used to calculate the background subtraction. *A* is the plateau AI defined by MBV with proportionality to unit volume of blood unit volume of tissue^−1^ and β is the rate constant of microvascular flow, units second^−1^ and proportional to MFV (Wei et al. 1998; Krix et al. 2005). Their product Aβ, with units of volume blood · volume tissue^−1^ · second^−1^, is proportional to MBF. Flow parameters were calculated in each individual, after background subtraction and graphing the average across three 28-sec flash-replenishment recording periods, by nonlinear regression using calculation of least-squares. In those subgroups undergoing multiple post feed CEUS measures, subsequent parameters were standardized and expressed as a proportion of the corresponding fasted value. The proportion of contrast-dependent AI recovered at B*t* was calculated from the difference between AI first recorded post flash (0.048 sec) and the mean of 10 subsequent measurements (over 0.48 sec) expressed as a fraction of the decrement seen on high MI flash.

### Statistical analysis

Analysis was via Prism 5.0b (Graphpad, San Diego, CA). Normality of distribution was tested using D'Agostino and Pearson omnibus normality test. Data are presented as mean + SE or when not normal distributed, as median + interquartile range. Comparisons between measures made at times before and after feeding were made by means of two-way repeated measure analyses of variance (ANOVA) with Bonferroni posttests to locate significance. Paired *t*-tests were used to compare AI in each individual before the second and third flash to determine consistency across the recording period, while unpaired *t*-test was used to assess demographic and anthropometric effects of age. The Wilcoxon signed rank test was used to determine if the proportion of bubbles burst by high MI flashes differed from 100%.

## Results

### Subject characteristics

Our volunteer demographics are summarized in Table 1. BMI was higher in older men (mean 25.6 vs. 23.6 kg m^−2^, *P* = 0.001). Appendicular skeletal muscle mass index (ASMI) did not differ significantly between age groups (8.18 vs. 8.06 kg m^−2^, *P* = 0.34). No individuals were sarcopenic as defined by Baumgartner et al. (1998) though lean leg mass was significantly less in older men 19.7 ± 0.6 versus 17.6 ± 0.4 kg, *P* = 0.004.

**Table 1:** Participant demographics; mean ± SEM.

	Young men	Older men	Young fed subgroup	Older fed subgroup
*N*	14	22	6	7
Age	20.4 ± 0.55	69.7 ± 0.62	19.8 ± 0.61	69.6 ± 0.81
BMI (kg m^−2^)	22.5 ± 0.38	25.6 ± 0.48*	22.3 ± 0.56	25.5 ± 0.44*
HGS (kg)	45.7 ± 1.90	40.1 ± 1.23	41.0 ± 3.16	40.7 ± 1.33
ASMI (kg m^−2^)	8.18 ± 0.13	8.06 ± 0.16	8.09 ± 0.12	8.15 ± 0.19
Lean leg mass (kg)	19.7 ± 0.59	17.6 ± 0.38**	18.6 ± 0.59	17.4 ± 0.41*

BMI, body mass index; HGS, hand grip strength assessed by dynamometry; ASMI, Appendicular skeletal muscle mass index (limb lean mass × height^−2^).

* *P* < 0.05 versus young.

** *P* < 0.01 versus young (unpaired *t*-test).

### Establishing a steady state of circulating Sonovue™ microbubbles

**Figure 2:**
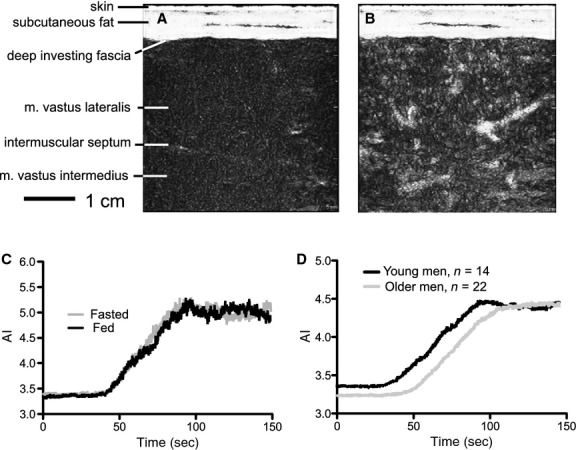
Ultrasound images recorded using contrast-specific scan mode (suppressing tissue derived signal) in one individual, (A) Before contrast infusion and (B) During steady-state microbubble circulation. AI (Acoustic intensity) increases with microbubble infusion. (C) One individual, changes in AI with microbubble infusion recorded on two occasions, before and after feeding. (D) Average change in intensity on microbubble infusion in fasted young (*n* = 14) and older (*n* = 22) men. Time after commencement of infusion.

**Figure 3:**
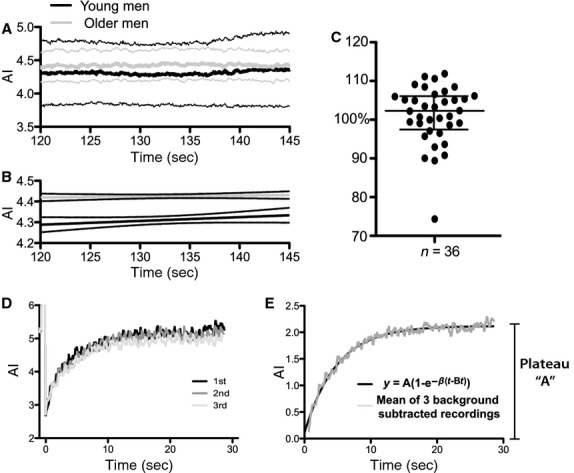
Steady state of circulating microbubbles is achieved prior to starting destruction/replenishment cycles and high MI “flash” burst approximately all microbubbles. (A) AI prior to starting destruction/replenishment recordings (mean ± 95% CI). (B) Linear regression analysis of AI/time curves from A. Gradients are 0.00182 sec^−1^ (from 95% CI −0.0007 to 0.004) in young and 0.000459 sec^−1^ (from 95% CI −0.0008 to 0.002) in older men. (C) Percent of signal, attributable to contrast microbubble backscatter, that is lost upon first flash, in fasted individuals. Time after commencement of microbubble infusion. AI/Time curves after high MI destruction flash follow an exponential association curve. (D) Three successive flash – replenishment cycles in one young individual, recorded over 90 sec, aligned by flash. (E) Background subtracted mean of three cycles fitted to an exponential association function. Time post high MI flash.

Upon infusion of Sonovue™, AI increased in every individual. Figure 2A and B show actual screen captures from one individual before and during Sonovue™ infusions. The increase in AI was the same across age groups (*P* = 0.67). Figure 2C and D show the change in quadriceps muscle AI during the first 2.5 min of infusion. No difference in AI of muscle was found between young and old men, either before (*P* = 0.72) or during (*P* = 0.90) microbubble infusion. Individuals reached a steady state of Sonovue™ circulation within ∼100 sec of commencement of infusion. The period 120–145 sec (Fig. 3A) was subject to linear regression analysis (Fig. 3B) to confirm constancy of echo signal, prior to the commencement of microvascular flow assessment at 150 sec. It was demonstrated that the slope was approximately zero in both age groups (mean dAI/d*t*, 0.00182 sec^−1^ in young and 0.000459 sec^−1^ in older men. Neither differed significantly from zero (*P* = 0.15 and 0.47, respectively). As there was no significant difference detected between lines, it was possible to calculate a pooled mean dAI/d*t*, of 0.000979 sec^−1^ equivalent to 1.3%/min. Further confirmation of continuation of steady-state throughout the recording period was derived from comparison of absolute, non background subtracted AI recorded immediately prior to the second and third flashes, thus 30 sec after the first and second flashes. AI was unchanged: mean 4.290 fasted versus 4.292 fed (*P* = 0.959).

### Confirming near-complete microbubble destruction

The decrement in AI following high MI insonation was expressed as a proportion of AI attributable to microbubbles (Fig. 3C). This ranged from 89 to 109% in young and from 74 to 112% in older men, with median values of 104% and 100%, respectively. Neither differed significantly from 100% (*P* = 0.19, 0.48). Thus, the energy delivered by the brief, high MI insonation “flash” was sufficient to disrupt approximately all the microbubbles in the field of view.

### Confirming exponential association

**Figure 4:**
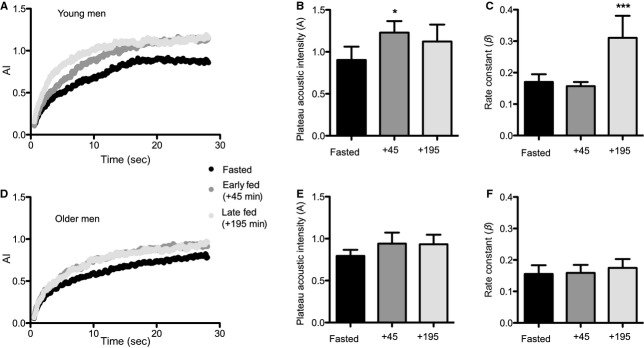
Muscle microvascular flow parameters derived from microbubble replenishment following high MI flashes show a vascular response to feeding in young that is lost in older men. Time after high MI flash. (A and D) show average replenishment curves measured in six young and seven older men before and at two times after feeding. (B) (young) and (E) (older) demonstrate the plateaus of exponential association curves fitted to individual replenishment curves, proportional to MBV (mean ± SEM). (C) (young) and (F) (older) show rate-constants of the same curves, proportional to MFV (mean ± SEM). *P = 0.012, ***P = 0.0004, versus fasted, RM 2-way ANOVA.

Change in AI observed in one individual over time following a high MI flash is shown in Figure 3D, with actual observed AI over three flash-replenishment cycles aligned by flash. Figure 3E shows background subtracted mean AI of three cycles. Post-flash AI fits the exponential function AI = A(1−e^−β(*t*−B*t*)^) where A is the plateau proportional to MBV, β the rate constant proportional to MFV, t the time post flash, and B*t* the time of background correction. This equation provided a good description of the relationship of AI to *t*, with median *R*^2^ (goodness of fit) = 0.927 and 0.946 in young and old, respectively. Background subtraction was performed using the mean AI between 0.048 and 0.520 sec post flash, B*t* was thus ∼0.28 sec while calculation of exponential parameters began 0.52 sec post flash. The use of this time window permitted subtraction of background AI equal to the sum of tissue-dependent AI plus early filling (Sjøberg et al. 2011). This accounted for up to 19% (median 11.9%) of total contrast-dependent AI in young men and up to 18% (median 9.1%) in older men. As the same fraction of vascular filling occurred during background correction in young and old men, age-specific compensations were not undertaken. Interindividual variability was assessed in 14 young and 22 older men. Coefficient of variation (CoV) of MBV was 37% in young and 39% in older men, respectively; CoV of MFV was ∼38% in both (Fig 4).

### Microvascular responses to feeding

**Figure 5:**
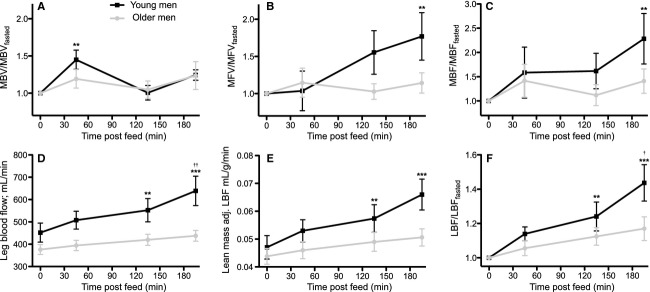
Vascular responses to feeding in muscle; a biphasic response in young men that is lost in older men. (A) Young men demonstrate an early increase in MBV reflecting capillary recruitment. (B) After a latency period, MFV increases in young men. (C) Only in young men does MBF increase with feeding. Mean ± SEM, all standardized to fasting. (D) Changes in leg blood flow are observed in young men with a temporal pattern in keeping with CEUS calculation of MBF. Femoral flow by Doppler US. (E) Femoral flow adjusted for lean leg mass (F) Adjusted femoral flow, each individual standardized to their own fasting flow. **P < 0.01 versus fasted, ***P < 0.001 versus fasted. ^†^*P* < 0.05 old versus young, ^††^*P* < 0.01 old versus young. Time after feeding. Leg flow measures (D–F) represent means of common femoral artery flow measured between 65 and 5 min pre feed (0) and at post feed intervals +5 to +65 min (45), +95 to +155 min (135) and +155 to +215 min (195).

We characterized the time course of vascular responses to oral EAA feeding. In younger men, a significant vascular response to feeding occurred manifesting as an early increase in MBV followed by a later increase in MFV (Fig. 5). Changes in microvascular flow parameters over time are shown standardized to fasting values (Fig. 5A–C). At 45 min post feed, MBV had increased 36% from fasting (*P* = 0.008, *n* = 6). At this time MFV and MBF were unchanged from fasting. However, at 195 min post feed, MFV had increased 77% (*P* = 0.007) and MBF 130% (*P* = 0.008) over fasting. In stark contrast, at no point did we observe any significant microvascular responses to feeding in older men; this strongly highlights an age-related blunting of postprandial microvascular recruitment. Small sample size and interindividual variability prevented demonstration of significant difference between age groups (MBV at +45 min, old vs. young, *P* = 0.13; MFV at +195 min, old vs. young *P* = 0.07).

### Whole-leg flow responses to feeding

Changes in femoral artery blood flow broadly followed those of muscle microvascular flow, Figure 5D–F. Although younger men exhibited increased femoral blood flow of 24% (*P* = 0.005) 135 min after feeding and 44% (*P* < 0.0001) 195 min after, at no time point were femoral flow changes increased over fasting in older men, thus demonstrating blunted macrovascular recruitment. Interindividual coefficients of variation, comparing averages across 60-min windows as shown in Figure 5, are 24–27% (young) and 26–29% (older). Intraindividual coefficients of variation (between 12 measures across the fasting period) were 3% (young) and 3.4% (older).

### Plasma Insulin

**Figure 6:**
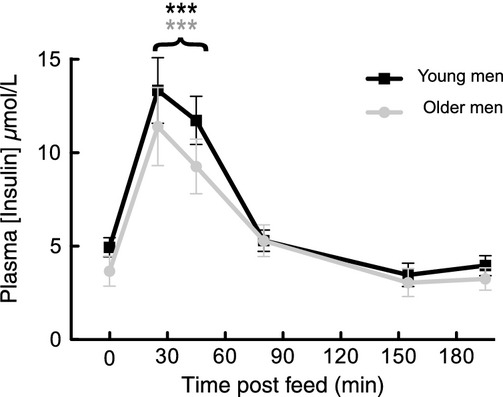
Plasma insulin response to 15 g EAA feeding. Time after feeding. ***P < 0.001 versus fasting. No significant difference observed between age groups.

This feeding protocol stimulated transient hyperinsulinemia (Fig. 6). Fasting plasma insulin concentrations were similar in young (4.1 ± 0.82 mU L^−1^) and older men (4.0 ± 0.52 mU L^−1^, *P* = 0.45). Insulin concentrations peaked 25 min after feeding in young (13.3 ± 1.8 mU L^−1^) and older groups (11.4 ± 2.1 mU L^−1^). By 45 min after feeding, insulin concentrations remained elevated in young (11.7 ± 1.3 mU L^−1^, *P* = 0.002 vs. fasting) and older men (9.3 ± 1.5 mU L^−1^, *P* = 0.004 vs. fasting). Serum insulin returned to fasting concentrations 80 min after feeding and remained unchanged from fasted throughout mid and late CEUS recordings. There were no differences between age groups.

## Discussion

We have developed and validated a novel short recording protocol using a widely available and clinically safe contrast agent, and revealed application in detecting subtle and temporal changes seen in muscle microvascular flow (e.g., in response to nutrition); we also provide specific details of an important physiological phenomenon, viz, an age-related blunted microvascular recruitment in response to a feeding stimulus, that is, oral 15 g EAA, which also acts to stimulate physiological insulinemia.

**Figure 7:**
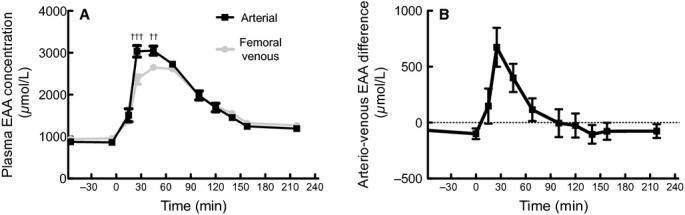
Essential amino acid (EAA) disposal estimated by arterio-venous differences in plasma concentration. (A) Venous and arterial plasma EAA concentrations in response to 15 g EAA feeding in men ∼70 years, *N* = 8. Mean ± SEM. ^††^*P* < 0.01, ^†††^*P* < 0.001 arterial versus venous. Measurements with Biochrom 30 AA Analyser (Cambridge, U.K.). (B) Arteriovenous difference in EAA concentration. Time after feeding.

We have provided the first description of the time course of muscle microvascular responses to feeding. We report that young men exhibit a bi-phasic response, with early increases in MBV reflecting capillary recruitment and later increases in MFV, MBF, and leg blood flow (LBF) reflecting dilatation of more proximal conduit vessels. Taken together, these findings demonstrate, at a microvascular level, a temporal dissociation between feeding-induced muscle capillary recruitment and changes in leg blood flow. Our observation of an early increase in MBV was at a time when plasma insulin was at its peak (30–60 min after feeding) and when LBF remained at fasted values. These findings are in keeping with insulin-induced nitric oxide (NO)-mediated capillary recruitment preceding increases in LBF in rodents (Vincent et al. 2002; Zhang et al. 2004). Furthermore, our findings support the notion that terminal arterioles rapidly respond to physiological hyperinsulinemia, and in a time scale that would facilitate both insulin/AA delivery to muscle to support muscle protein anabolic and insulin-mediated actions on glucose uptake processes. Amino acid disposal is maximal at 45 min post EAA ingestion supporting the physiological relevance of capillary recruitment at this time (Fig. 7).

Our next observations of a delay between maximal MBV (<1 h post feeding) and maximal LBF, MFB, and MFV (>3 h post feeding) invites speculation regarding mechanisms that account for such differences. Insulin's vascular actions are mediated via antagonistic NO-mediated vasodilatation (Steinberg et al. 1994) and the 21-AA polypeptide, endothelin-1 (ET-1)-mediated vasoconstriction. Net vasodilation in terminal arterioles would achieve capillary recruitment, increasing nutrient delivery as described above. Maintenance of tone, by balanced ET-1 signaling in more proximal vessels would offset changes in LBF and thus prevent a postprandial drop in systemic vascular resistance and blood pressure. Thus, a scenario of balanced, antagonistic vasoactive molecules, facilitating muscle exchange while minimizing systemic impact, may be at play after eating, similar to that proposed to exist during exercise (Barrett-O'Keefe et al., 2013a). Circulating ET-1 concentrations rapidly elevate with hyperinsulinemia (Wolpert et al. 1993; Ferri et al. 1995) though there is a paucity of evidence for ET-1 signaling, in vivo, beyond 2 h post insulin stimulation and human prepro-ET-1 mRNA is labile with an intracellular half-life of ∼15 min (Inoue et al. 1989). Thus, the latency before increases in MBF and LBF may be due to persistence of vasodilatory stimuli beyond that of the antagonistic vasoconstrictors. Alternative explanations could include endothelium-dependent hyperpolarization of vascular smooth muscle of more proximal resistance arteries and subsequent Ca^2+^ depletion contributing to gradual onset dilatation (McGuire et al. 2001; Hayflick 2007; Feletou 2009) or more slowly released venule-derived factors, such as prostanoids (McKay et al. 1998).

Given our previous observation that the “active” duration of muscle protein anabolism is <3 h, the extended latency of LBF/MBF changes calls into question their physiological relevance – at least in terms of protein anabolism (Atherton et al. 2010; Bohe et al. 2001). Increased flow velocities shift exchange vessel blood characteristics toward those of arterial blood and thus promote exchange of molecules with a large arteriovenous difference. The arteriovenous difference in plasma EAA concentration seen in response to a 15 g EAA feed, maximal at 45–65 min post feed, is lost by 120 min. Thus, at least with this feeding strategy, increases in MVF (the predominant driver of increases in MBF and following that in LBF) do not appear to facilitate AA delivery, uptake or subsequent muscle protein synthesis. Importantly, for the first time we have also shown that both MBV and MVF components of postprandial muscle microcirculatory response are compromised with aging, as was LBF (the latter was previously reported by us [Phillips et al. 2012]). On this basis, we conclude that marked diminutions in postprandial muscle microvascular recruitment (particularly MBV) in old versus young men may contribute to impaired muscle glucose disposal and AA utilization for protein synthesis(Cuthbertson et al. 2005); factors implicated in development of metabolic disease and sarcopenia (Mitchell et al. 2012).

Key physiologically insightful benefits of our newly validated Sonovue™ technique exist. Indeed, while limb “bulk” flow reflects both nutritive and nonnutritive routes, CEUS permits selective study of slower flowing nutritive vessels by disregarding signal derived from the fastest filling non exchange vessels (10% of vessel filling occurred by 0.28 sec and these vessels were excluded from subsequent calculations). Moreover, the usefulness of techniques that assess whole-limb bulk blood flow as a proxy for muscle nutrient flow is called into question by our observation that the magnitude of increase in muscle perfusion in the young (>100%) was much greater than the corresponding increase in femoral arterial flow (<50%) indicating rerouting of blood within the limb. Even more significantly, muscle capillary recruitment occurs without detectable changes in femoral artery flow. There is also a complex relationship between perfusion and exchange that is, despite simultaneously increasing the exchange area and reducing the distance from capillary to myocyte (Krogh 1919), if accompanied by a reduction in MFV, overall perfusion may not change. As our technique is able to measure MBV, MFV, and therefore MBF, the relative contribution of these indices can be accounted for. Thus, the ability of CEUS to exclude the fastest filling, non exchange vessels and resolve separate components of perfusion make it a powerful, noninvasive technique in assessing the microcirculation of skeletal muscle and potentially other vital organs. We contend that determining muscle blood flow and its components will best facilitate research into muscle microvascular perfusion; approaches not easily achieved by conventional methods for example, US Doppler, thermo-dilution or venous occlusion plethysmography. While this study explores microvascular responses, we cannot comment on the impact of these responses upon nutrient handling as age-related differences, for example, in amino acid disposal, may reflect other metabolic features of senescence.

The use of Sonovue™ microbubble in the application of quantitative CEUS offers several benefits. Using a primed constant infusion protocol a steady state of circulating microbubbles can be achieved within two half-lives, thus reducing the time taken before measurement of flow parameters by 75% compared to Sjøberg et al. (2011). A short infusion protocol facilitates the use of this technique in situations where flow changes are short lived, including after exercise and in response to disruption of arterial inflow or to changes in systemic hemodynamics. For example, recordings can be completed within 100 sec of exercise cessation. Previous studies that have employed short unprimed infusion protocols have been subject to unstable contrast concentrations. Krix et al. describe an ongoing increase of 1.2% sec^−1^ at the start of recordings, almost 60 times what we observed (Krix et al. 2009). Changing microbubble concentration has significant potential to compromise accuracy in calculation of flow parameters. Sonovue™ also has a long shelf life (∼2 year) and can be stored at room temperature. No specialist equipment is required to reconstitute Sonovue™ at the time of administration. There is no recognized safe maximal dose with product literature documenting Phase 1 trial administration of 56 mL without adverse effects. This has allowed us to perform four serial CEUS assessments and thus characterize the time course of vascular responses. Perflutren lipid microspheres (e.g., Definity™, Lanthus Medical imaging, North Billerica, MA), the only other agent to date used to assess changes in skeletal muscle blood flow in response to stimuli other than exercise, requires refrigeration prior to use and reconstitution is only via a specialized oscillator. Furthermore, a safe upper dose of 3.6 mL/24 h-period exists, limiting multiple repeated recordings. Perflutren-based contrast agents are not currently available commercially in Asia and have been unavailable in Europe since 2008. Sonovue™ is not currently available in the United States and issues of contrast availability continue to hamper research in the field. Levovist™ (Schering AG, Berlin, Germany, air-filled microbubbles stabilized by palmitic acid) exhibits low harmonic behavior and high MI destruction is needed to differentiate contrast from tissue (Blomley et al. 1998; Quaia et al. 2002).

Some limitations do exist to the use of this technique. High interindividual variability in contrast-dependent AI compromises the ability of CEUS to measure differences between subjects, with observed CoV of A and β of 35–40%. Therefore, and in keeping with previous studies able to detect subtle (<100%) changes in flow characteristics this study has been completed within individuals undergoing repeated assessments on the same day with recording equipment staying in-situ throughout and we have not measured intraindividual day-to-day variation.

The parameter “A” has proportionality to MBF but it is an index not an absolute value; interindividual variation in distribution volume and elimination of infused microbubbles will change the measured A value for a given MBF.

**Figure 8:**
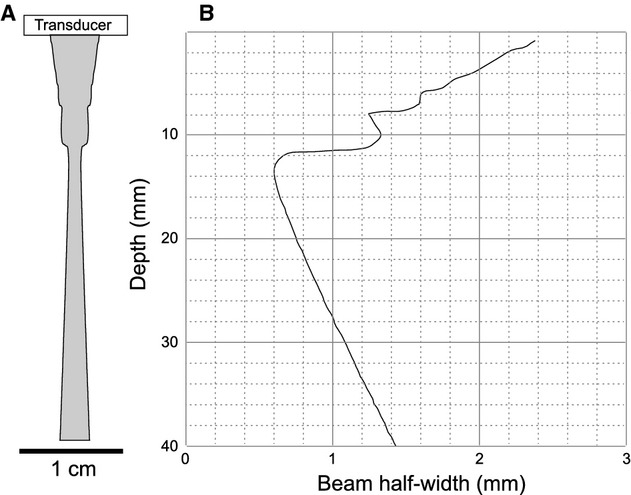
Ultrasound beam width depends upon distance from transducer, transmission frequency, tissue characteristics, and focusing technology. Intensity decreases with distance from the central axis. (A) example of a 2-D cross section of a beam modeled on the distance from the central axis at which intensity has dropped to 50%. A reduction in the beam intensity will provide insufficient energy to achieve microbubble disruption. (B) beam half-width against depth showing the −3 dB width (consistent with 50% reduction in intensity). Images courtesy of Steve Jasiok, Phillips Healthcare.

**Figure 9:**
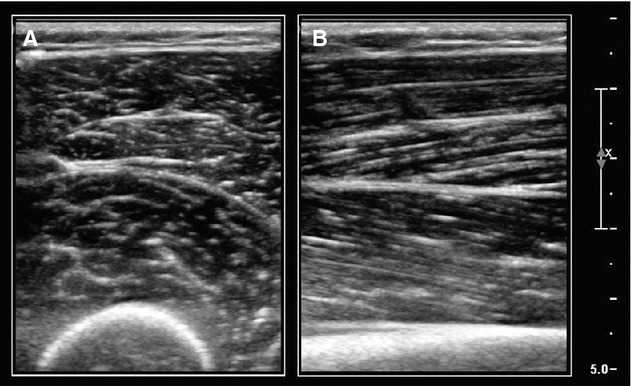
Muscle fascicle orientation in the mid thigh visualized using B-mode ultrasound. (A) coronal plane, perpendicular to long axis of leg as used for CEUS recordings, (B) sagittal plane view of the same region demonstrates *penate* nature of quadriceps muscles; fascicles are orientated at an angle to the long axis of the muscle.

Moreover, as “β” is a vector quantity, its measurement of is dependent upon the shape of the ultrasound beam and, due to the muscle microvascular anatomy previously described, the angle of the transducer to the long axis of the muscle fibers. Figure 8 presents a beam profile representative of that used in this study. During theoretical development of these techniques the assumption was made that the beam propagated through the tissue with constant sized, rectangular cross section (Wei et al. 1998; Sjøberg et al. 2011). In reality the ultrasound beam undergoes divergence as it moves away from the transducer as shown here. Moreover, the shape of the beam changes with transmitted frequency and, when using a multi-crystal transducer, can be manipulated by changing the temporal pattern of pulsing of crystals to create a focal zone at a defined distance along the central beam axis. Constructive interference in this zone, between in-phase waves from different parts of the transducer, serves to increase intensity and to minimize effective beam width. Thus, a beam “waist” exists at a variable depth with a wider beam above and below (Quaia 2005; Tole 2005). The intensity of the beam decreases with distance from the mid axis. Assumption of a uniform beam thickness will tend to underestimate the MFV of muscle close to the beam waist, while overestimating the MFV of muscle above and below it. Figure 9 provides a B-mode ultrasound image of the region assessed. As described previously, muscle microvascular anatomy is not random but highly axial, with capillaries running in the same orientation as muscle fibers. As β estimates MFV as a vector, units beamwidths · sec^−1^ (Krix et al. 2003), it will be dependent upon the angle of the US transducer to the long axis of the muscle fascicles. MFV will be maximal when perpendicular to the fibers. Our transducer housing maintained an orientation in the coronal plane on the thigh, close to and perpendicular to the fibers of VL. The range of angles of pennation of VL, seen with atrophy and hypertrophy (Narici and Cerretelli 1998), will influence comparison between individuals. As *A* estimates MBV as a directionless quantity it will be independent of the orientation of transducer in relation to muscle.

To conclude, we present and apply a new and novel CEUS protocol, which has the ability to resolve complex changes in human muscle microvascular flow. We use this technique to demonstrate a biphasic response to amino acid ingestion and reveal an early postprandial capillary recruitment phase that is followed by later increases in limb muscle bulk flow. We propose that the capillary recruitment phase should serve to facilitate nutrient delivery under physiological conditions. We also demonstrate a marked attenuation of these responses in older men, perhaps impacting upon insulin sensitivity, glucose disposal and the maintenance of healthy muscle mass.

## Clinical Trial Registry

Trial Registry: ClinicalTrials.gov:

Registration Number: NCT01735539

URL: http://www.clinicaltrials.gov

## Supporting Information

**Video S1.** Microbubble destruction/replenishment cycle following high MI flash in a young fasted individual.
